# Injectable pH-responsive hydrogel for combinatorial chemoimmunotherapy tailored to the tumor microenvironment

**DOI:** 10.1186/s12951-022-01561-z

**Published:** 2022-08-11

**Authors:** Jun Gu, Gang Zhao, Jiangkun Yu, Pei Xu, Jiabin Yan, Zhengshuai Jin, Sheng Chen, Yong Wang, Leshuai W. Zhang, Yangyun Wang

**Affiliations:** 1 State Key Laboratory of Radiation Medicine and Protection, School for Radiological and Interdisciplinary Sciences (RAD-X), Collaborative Innovation Center of Radiation Medicine of Jiangsu Higher Education Institutions, Soochow University, Suzhou, 215123 China; 2 The Affiliated Jiangsu Shengze Hospital of Nanjing Medical University, Suzhou, 215228 China

**Keywords:** Injectable hydrogel, pH-responsive, Silk-chitosan composite, Chemoimmunotherapy, Tumor microenvironment

## Abstract

Although combination chemoimmunotherapy shows promising clinical results for cancer treatment, this approach is largely restricted by variable objective response rate and severe systemic adverse effects of immunotherapeutic antibody and chemotherapeutic drugs. Therefore, an in situ-formed therapeutic silk-chitosan composite scaffold is fabricated in this study to allow local release of the chemotherapeutic drug doxorubicin (DOX) and JQ1 (small molecular inhibitor used for the extraterminal protein BRD4 and bromodomain) with control release kinetics. DOX-JQ1@Gel contains a pH-degradable group that releases therapeutics in a weak acidic tumor microenvironment. The released DOX could directly kill tumor cells or lead to immunogenic cell death, thereby triggering the response of antitumor immunity. Meanwhile, chemotherapy-triggered antigen release and JQ1-mediated PD-L1 checkpoint blockade cumulatively contribute to trigger the response of antitumor immunity. Finally, the DOX-JQ1@Gel is locally injected to evaluate its synergistic cancer therapeutic effect, which is expected to improve objective response rate of immunotherapy and minimize systemic side effects.

**Graphical Abstract:**

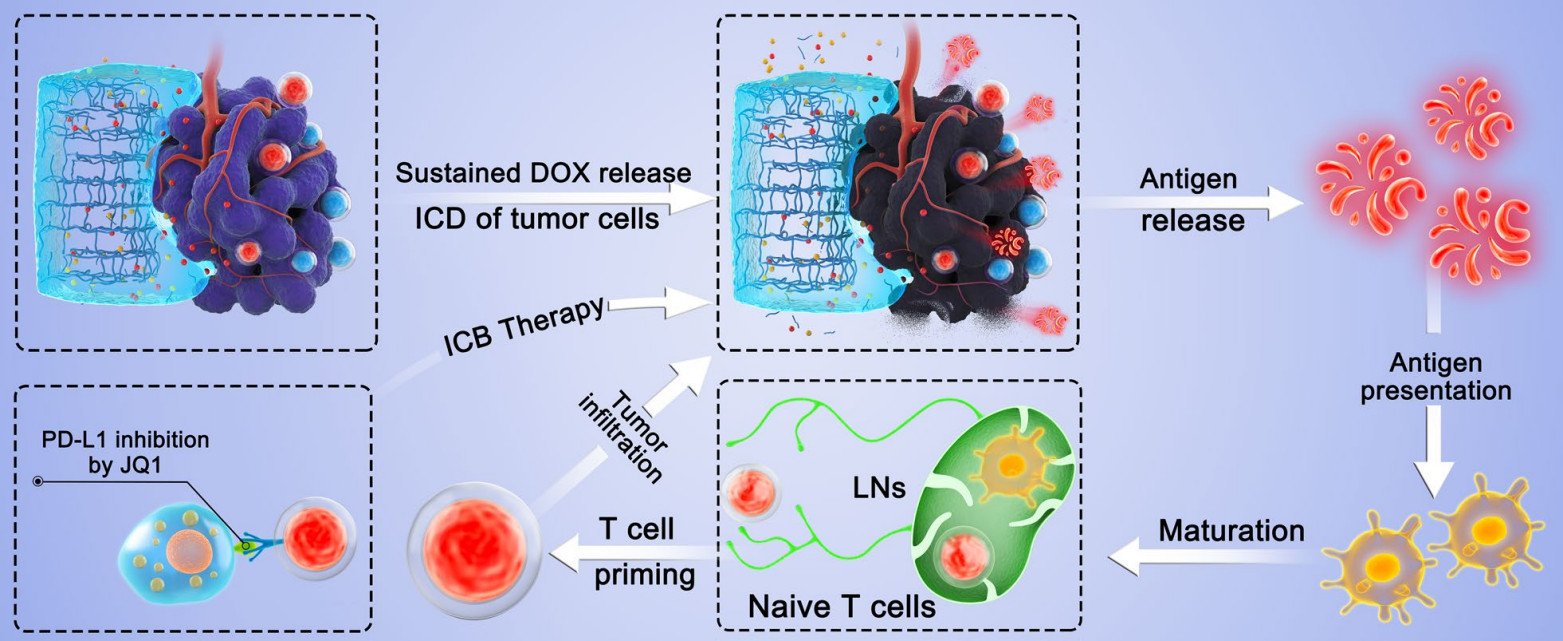

**Supplementary Information:**

The online version contains supplementary material available at 10.1186/s12951-022-01561-z.

## Introduction

Immunotherapy has attracted considerable research attention and significant progress has been made for cancer therapy in the past decade [1]. Significant clinical responses are induced by immune checkpoint blockade (ICB) oriented toward the cytotoxic T lymphocyte-associated antigen 4 or programmed death 1 (PD-1)/programmed death ligand 1 (PD-L1) pathway in different malignancies, such as nonsmall cell lung, melanoma, kidney, bladder, neck, and head cancers, in clinically approved immunotherapy approaches [2]. More than six PD-1/PD-L1 pathway-inhibiting antibodies have been applied to the treatment of over 10 categories of cancers by 2020 since the United States Food and Drug Administration approved the first ICB antibody (ipilimumab) for blocking the CTLA-4 pathway in treating metastatic melanoma in 2011 [3]. Despite its success in clinical application, the variable objective response rate against different tumor categories and serious immune-related adverse events after systemic delivery limits ICB therapy [4]. Reports showed that only ~ 20% of patients benefit from the ICB treatment [5]. Thus, exploring strategies to increase ICB response and avoid serious side effects is necessary.

Local administration and regulated release of immunotherapeutic agents can avert side effects related to checkpoint inhibitors for efficient systemic administration as well as improve treatment efficacy [6]. In addition, side effects can be avoided more efficiently with local administration than intravenous injection, which is a conventional injection mode to treat cancer [7]. For example, Chen et al. created a sprayed fibrin gel for local and sustained delivery given that thrombocytopenia and anemia caused by the antibody anti-CD47 must be reduced [8]. Various types of immunotherapeutic agents, such as antibodies, small-molecule drugs, and even cells, are locally administered to enhance cancer immunotherapy, which demonstrates minimal immune-related side effects [8–15].

Clinical evaluation of immunotherapy is mainly focused on single agents that target individual steps in the host antitumor immune response; meanwhile, the difficulty in solving primary mechanisms via monotherapies hinders the antitumor immunity of patients; hence, induction, potency, and persistence of host immune responses suggest the complicated interaction of different immune-cell populations with progressive tumors [16]. The unique tumor microenvironment can promote tumor progression and metastases and result in the resistance of tumors to different therapies [17, 18]. Notably, conventional chemotherapeutic drugs can lead to immunogenic cell death (ICD) of cancer cells and trigger certain degrees of antitumor immune responses caused by tumor-associated antigens in cancer cell debris when treated with chemotherapy [19–24]. A special type of anticancer drug involving doxorubicin (DOX) and gemcitabine presents modest activity when used as single treatments but can induce cancer ICD and efficiently enhance the immune response rate of ICB treatments [25].

**Scheme 1:**
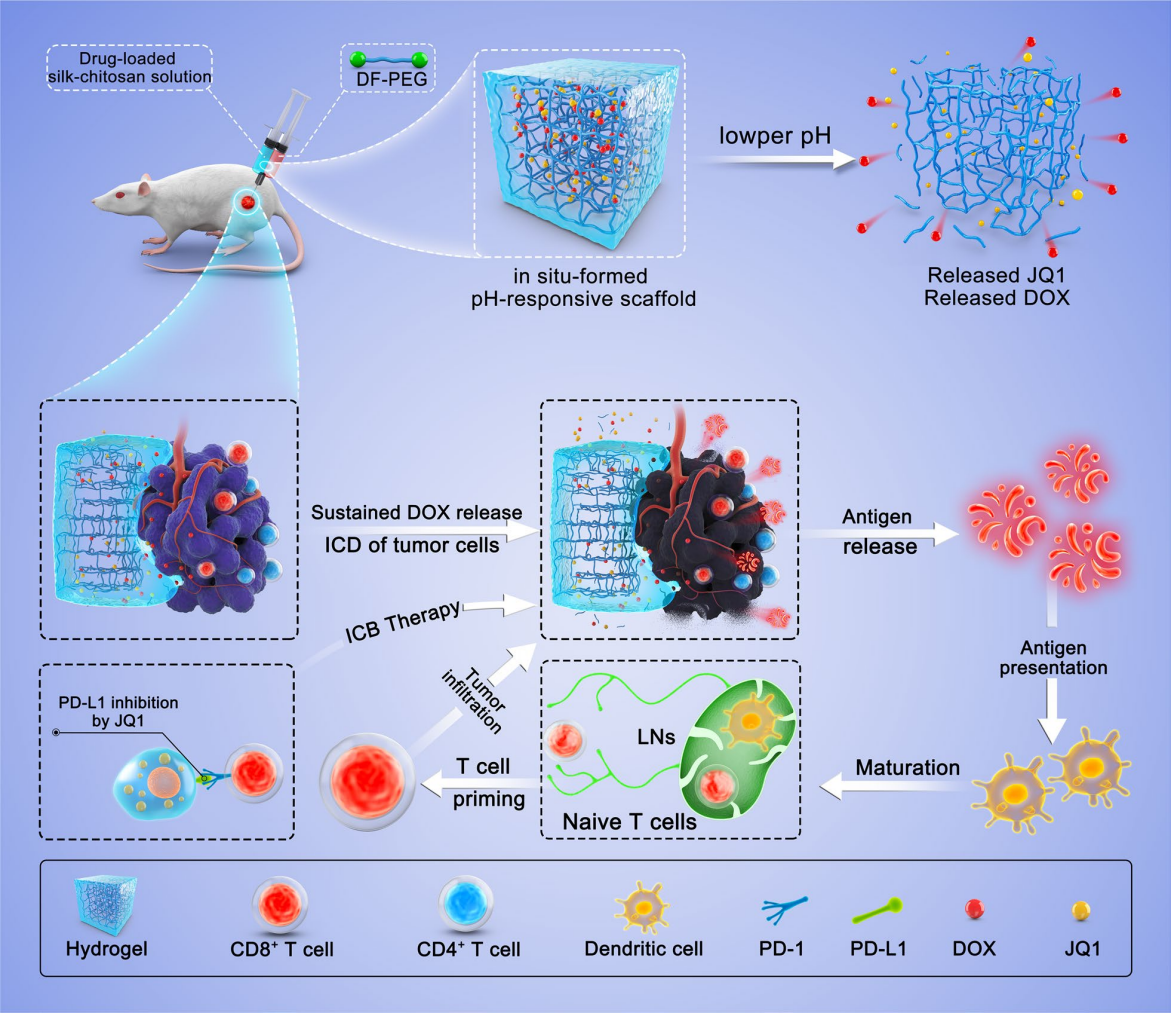
Schematic of combination chemoimmunotherapy using a pH-degradable hydrogel scaffold to deliver DOX and JQ1 into the TME

On the basis of these studies, we developed a DOX- (ICD inducer) and JQ1- (ICB inhibitor) loaded pH-responsive silk–chitosan composite hydrogel (DOX-JQ1@Gel) for combinatorial chemoimmunotherapy tailored to the tumor microenvironment (Scheme 1). Recent studies have shown that JQ1 (a small molecular inhibitor for extraterminal protein BRD4 and bromodomain) restrains the expression of IFN-γ-triggered PD-L1 during the transcription process; this finding indicated that JQ1 can potentially inhibit T lymphocyte-induced PD-L1 upregulation and later immune evasion [26–29]. Some advanced nanocarriers have been designed to deliver DOX and JQ1 on demand to tumor sites [30, 31], especially, local tumor injection was expected to further improve drug availability. For example, Zhao et al. developed a ROS-responsive hyaluronan-modified polydopamine nanoarray for delivering DOX/JQ1 and realizing immunosuppressive tumor microenvironment modulation and targeting therapy [30]. Based on these insights, DOX-JQ1@Gel was herein fabricated by integrating chemotherapeutics DOX and JQ1 into an acid-liable silk–chitosan composite hydrogel platform after intratumor injection. Moreover, the gradual release of DOX from the DOX-JQ1@Gel is triggered by the weak acid microenvironment and directly kills tumor cells and evokes the antitumor immune response by ICD. Meanwhile, the DOX-JQ1@Gel can potentially reduce IFN-γ-induced adaptive immune resistance by suppressing the transcription of PD-L1 with JQ1 and minimize side effects. Finally, the synergistic cancer therapeutic effect was assessed through the local injection of the DOX-JQ1@Gel, which is expected to improve the objective response rate of immunotherapy and minimize systemic side effects.

## Results

### Synthesis and characterization of in situ formed pH-responsive DOX-JQ1@Gel

**Fig. 1:**
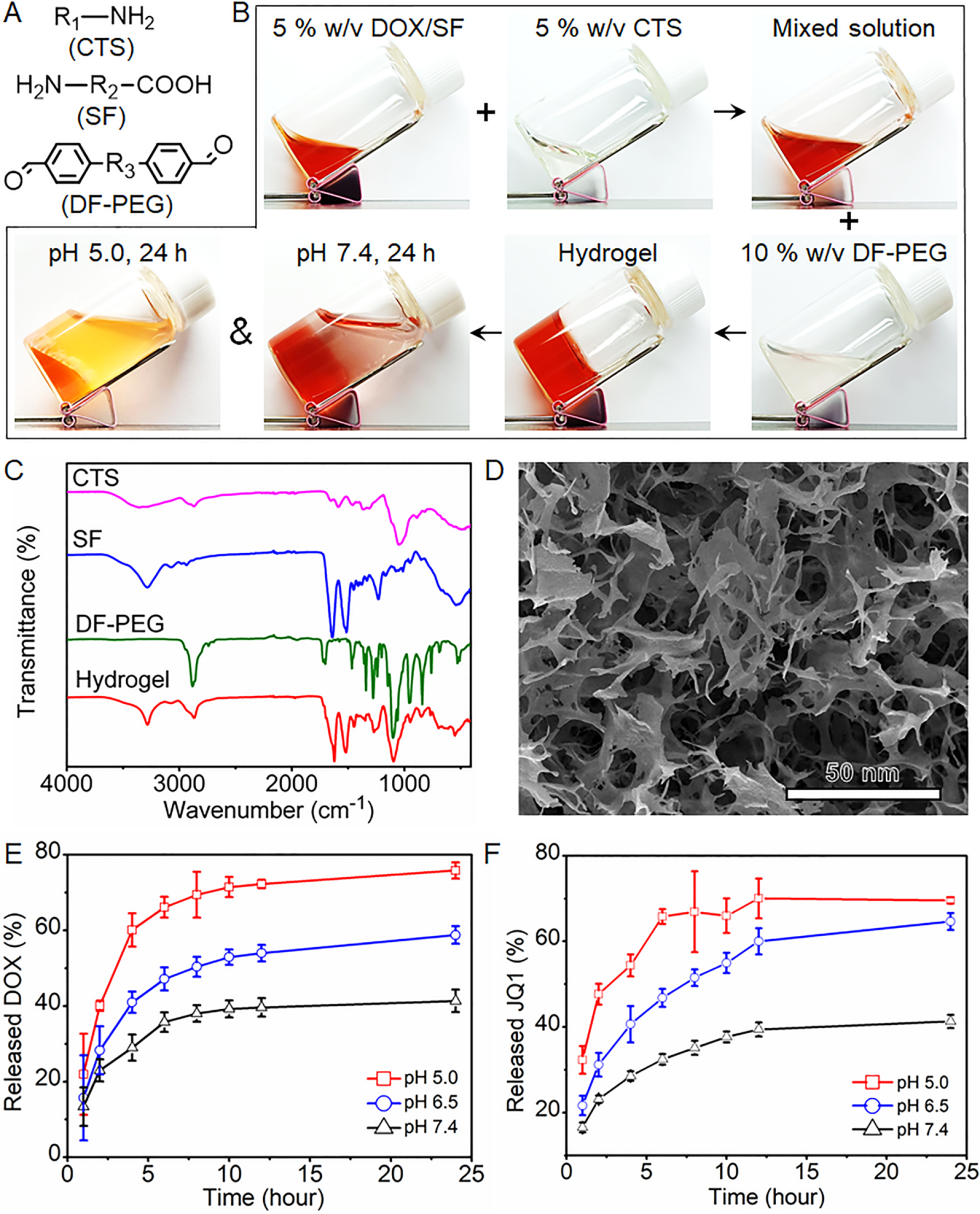
Characterization of pH-responsive hydrogel scaffold. **A** Simplified structure of chitosan (CTS), silk fibroin (SF), and dibenzaldehyde-functionalized polyethylene glycol (DF-PEG). **B** The formation process and pH-responsive property of hydrogel scaffold. DOX was used as a model drug and indicator. **C** Fourier transforms infrared spectroscopy (FTIR) of CTS, SF, DF-PEG and hydrogel. **D** Representative Cryo-SEM image of gel scaffold treated by pH 7.4 buffer solutions. **E**, **F** Cumulative release profiles of DOX and JQ-1 from hydrogels incubated with pH 7.4, pH 6.5 and pH 5.0 buffer solution, respectively

A typical Schiff base was chosen as the pH-labile linker on the basis of amine groups on silk–chitosan molecules to obtain pH-responsive injectable silk–chitosan composite hydrogel [32]. Dibenzaldehyde-functionalized polyethylene glycol (DF-PEG) was further prepared through esterification of hydroxyl-terminated PEG with 4-formylbenzoic acid. As shown in Fig. 1A, two aldehyde groups of DF-PEG can react with amine groups on the silk-chitosan mixed solution to form the Schiff base. The benzene ester carbonyl of silk-chitosan may enhance the stability of Schiff bases and accelerate crosslinking between DF-PEG and silk-chitosan to obtain gel networks. The in vitro gel formation process presented in Fig. 1B was examined in a transparent glass bottle. The gel was nearly degraded completely and DOX was visibly released after 24 h of incubation in the pH 5.0 solution. However, the swollen gel only caused a small amount of drug to leak even after 48 h of incubation at pH 7.4, as shown in Additional file 1: Fig. S1. This finding indicated that the loaded drug can be released from the biodegradable gel under acidic condition. The results of Fourier transform infrared spectroscopy (FTIR) shown in Fig. 1C also demonstrated that the gel is successfully synthesized from the characteristic fingerprint region of 1500–500 cm^−1^. The results of scanning electron microscopy in Fig. 1D showed that the gel is a three-dimensional porous microstructure while the gel structure appears broken and pores become smaller after treatment with pH 5.0 buffer (Additional file 1: Fig. S2). DOX and JQ1 were co-encapsulated into the silk–chitosan scaffold named DOX-JQ1@Gel at therapeutically relevant doses (DOX, 5 mg/kg; JQ1, 5 mg/kg). Rheological properties of drug-loaded scaffolds are similar to those of empty ones, thereby indicating that the hydrogel formation remained unaffected by the drug encapsulation. The pH-responsive releasing profile of DOX shown in Fig. 1E is similar as the released JQ1 profile (Fig. 1F), resulting from the co-encapsulation capacity of DOX and JQ1. The release of JQ-1 and DOX can reach 80% in the solution with pH 5.0 over time while the release amount is only 50% and 40% in solutions with pH 6.5 and pH 7.4, respectively. These results indicated that the DOX-JQ1@Gel can be hydrolyzed when exposed to low pH values in the tumor microenvironment. This exposure caused the release of payloads and dissociation of the polymeric scaffold.

### DOX-JQ1@Gel shows immuostimulation abilities

**Fig. 2:**
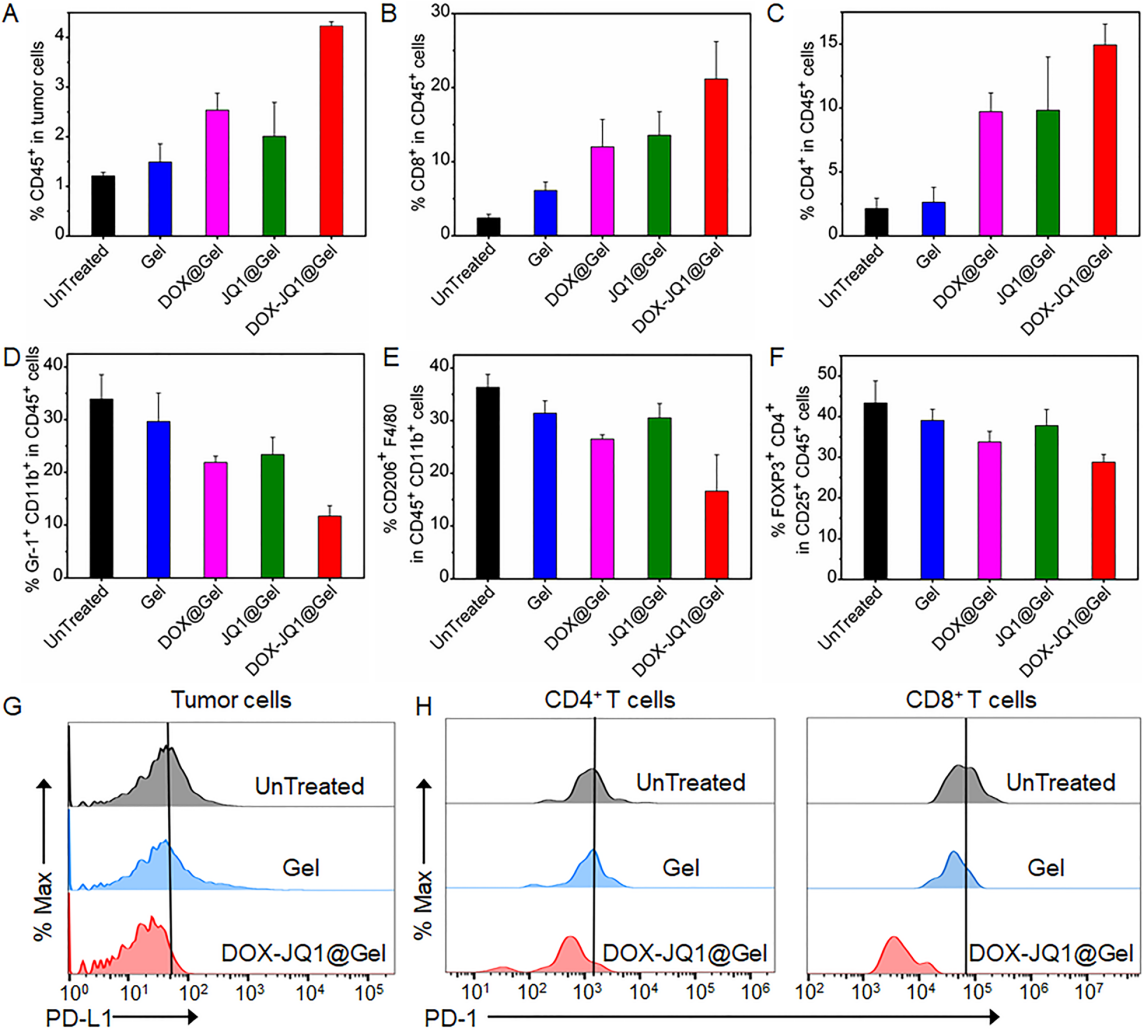
Local gel scaffold for eliciting immunogenic tumor phenotypes. 4T1-Luc tumors harvested from mice implanted with empty hydrogels, DOX@Gel, JQ1@Gel or DOX-JQ1@Gel were analyzed by flow cytometry 2 days after treatment. **A** Corresponding quantification analysis of T cell infiltration within the tumor. **B** Corresponding quantification analysis of CD8^+^ T cell infiltration within the tumor. **C** Corresponding quantification analysis of CD4^+^ T cell infiltration within the tumor. **D** Corresponding quantification analysis of MDSCs (CD11b^+^Gr-1^+^), gating on CD45^+^ cells. **E** Corresponding quantification analysis of M2 macrophages (CD206^+^) in F4/80^+^ CD11b^+^ CD45^+^ cells. **F** Corresponding quantification analysis of regulatory T cells (Tregs, FOXP3^+^ in CD4^+^ CD25^+^ CD45^+^ cells). **G** PD-L1 expression of tumor cells after empty hydrogel or DOX-JQ1@Gel treatment. **H** PD-1 expression of CD4^+^ and CD8^+^ T cell after empty hydrogel or DOX-JQ1@Gel treatment

To achieve in vivo application, the cytotoxicity of empty gel is firstly evaluated as show in Additional file 1: Fig. S3. After the normal cells (3T3) and breast cancer 4T1 cells were treated with different concentrations of raw materials, gels and gel extracts, the cell viability was all above 80%, which was beneficial to in vivo experiments. The effective retention in the tumor is beneficial to the sustained release of the drug in the tumor microenvironment [31]. In vivo biodegradability of DOX-JQ1@Gel was further verified by real-time fluorescence imaging and quantitative analysis shown in Additional file 1: Fig. S4. The in vivo tumor immune response of DOX-JQ1@Gel was next investigated in a 4T1 tumor-bearing BALB/c mouse model. Empty hydrogel (Gel), DOX@Gel, JQ1@Gel or DOX-JQ1@Gel was intratumorally (i.t.) injected into the 4T1 tumor xenograft when the tumor volume reached 100 mm^3^. After 1 week of treatment, tumors were excised and comminuted to form homogenates. The proportion of various immune cells in the homogenates were measured by flow cytometry shown in Additional file 1: Fig. S5, including tumor infiltrating lymphocytes (TILs, CD45^+^), CD8^+^ T cells, CD4^+^ T cells, myeloid-derived suppressor cells (MDSCs, Gr-1^+^CD11b^+^), tumor-associated M2 macrophages (M2-polarized TAMs, CD206^+^F4/80^+^) and regulatory T cells (Tregs, FOXP3^+^CD4^+^). As shown in Fig. 2A, it is found that DOX-JQ1@Gel notably increased the frequency of CD45^+^ TILs in the tumor microenvironment compared with empty hydrogel, DOX@Gel, and JQ1@Gel. The respective proportion of CD4^+^ and CD8^+^ T cells in Fig. 2B, C naturally activated by DOX-JQ1@Gel was approximately 1 and 1.5 times higher than the control group (DOX@Gel and JQ1@Gel). Figure 2D, E show a significant reduction of MDSCs and M2-polarized TAMs, while the change in Tregs shown in Fig. 2F is insignificant. To sum up, these results suggested that the DOX-JQ1@Gel increases the rate of TILs and reduces cellular components with immunosuppressive ability and chemotherapy and immunosuppressive agents can synergistically regulate the tumor immune microenvironment. The treatment of 4T1 tumor cells with DOX can cause cell death and inevitably increases the expression of PD-L1 and other signaling molecules, while that with JQ1 can inhibit PD-L1 expression in surviving cells. As shown in Additional file 1: Fig. S6, related indicators in vitro were evaluated, including ATP and calreticulinn (CRT), the expression of ATP and CRT gradually increases, indicating that immunogenic death is concentration-dependent. DOX-JQ1@Gel reduced the expression of PD-L1 in tumor cells compared with tumors treated with empty hydrogels (Gel) and untreated tumors (UnTreated) (Fig. 2G) as well as the expression of PD-1 in both CD8^+^ and CD4^+^ T cells (Fig. 2H). In addition, detection of circulating cytokines was conducted on the 4T1 tumor xenograft treated with Gel, DOX@Gel, JQ1@Gel, or DOX-JQ1@Gel. As shown in Additional file 1: Fig. S8A, B, interleukin-6 (IL-6) and interferon-γ (IFN-γ) were significantly upregulated after DOX-JQ1@Gel implantation and IFN-γ causes the expression of PD-L1 in tumor cells. Furthermore, lymphocytes of DOX-JQ1@Gel-treated mice tended to the spleen and induced a systemic antitumor immune response. Additional file 1: Fig. S9 illustrates that the number of cytotoxic CD8^+^ T cells in the spleen of DOX-JQ1@Gel-treated mice is doubled compared with the untreated group. These results confirmed that DOX-JQ1@Gel could lead to a robust T cell-mediated immune response in the microenvironment with tumor.

### DOX-JQ1@Gel-mediated combination therapy

**Fig. 3:**
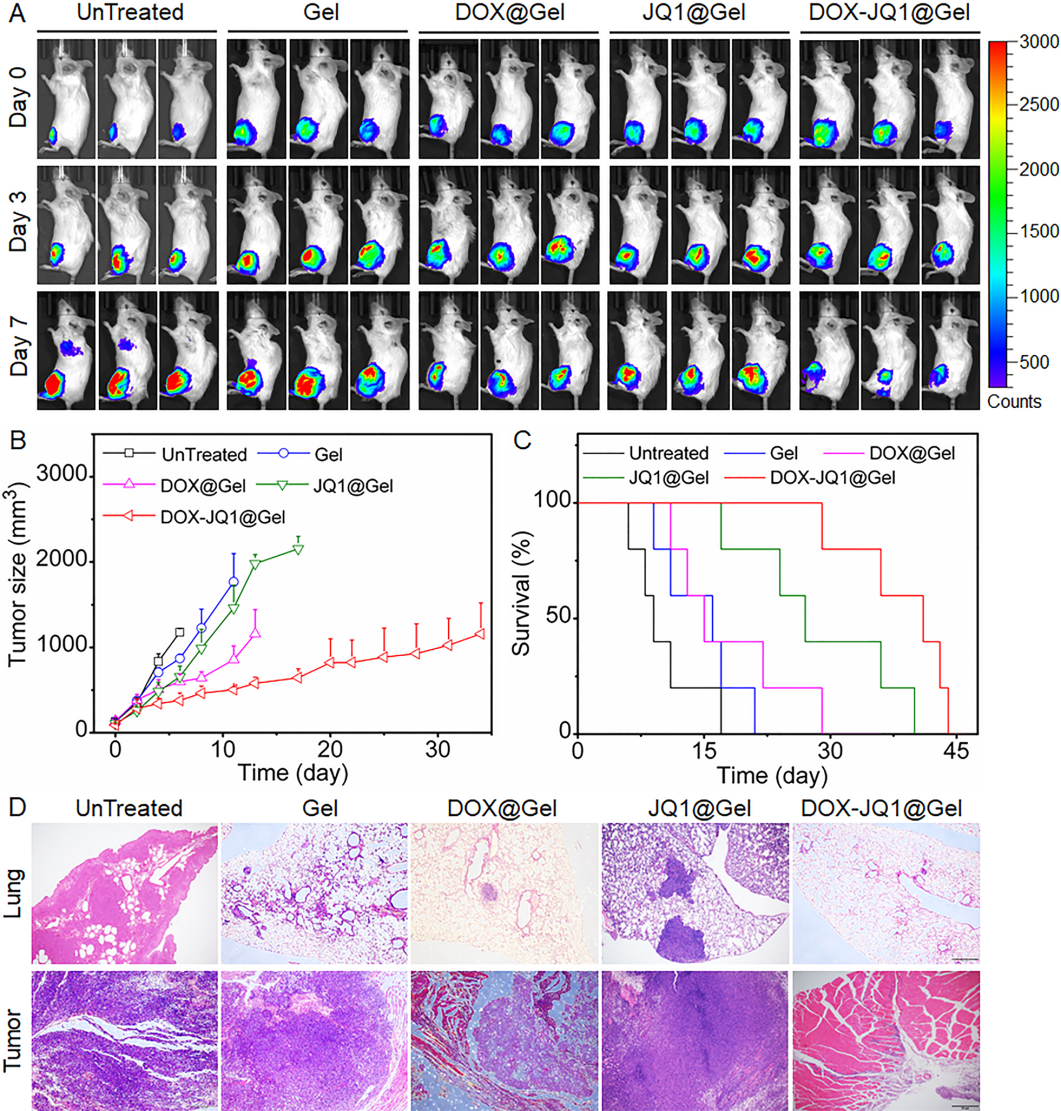
Local gel scaffold for inhibition of 4T1-Luc breast cancer growth in vivo. **A** In vivo bioluminescence imaging of the 4T1-Luc breast cancer in control and treated groups. Three representative mice of 5 mice per treatment group are shown. **B** Average tumor growth kinetics in control and treated groups. Growth curves represent means ± SEM; growth curves were stopped when the first animal of the corresponding group died. **C** Survival curves for the treated and control mice (*n* = 5). **D** Pathological H&E staining of lung and tumors from the treated and control mice at day 7 after treatments

Gel, DOX@Gel, JQ1@Gel, or DOX-JQ1@Gel was peritumorally implanted in 4T1-Luc tumor-bearing mice to verify if the proposed combined chemoimmunotherapy strategy could accelerate antitumor effects. As shown in Fig. 3A, bioluminescence signals of 4T1-Luc cells were used to monitor the tumor growth. The empty Gel suggested similar effects to those without treatment. The tumor growth in DOX@Gel- and JQ1@Gel-treated mice was delayed. By contrast, mice treated with DOX-JQ1@Gel showed significant tumor inhibition effects (Fig. 3B). Tumor sizes in mice were related to their survival (Fig. 3C). Among the mice, 80% survived a minimum of 30 days after treatment with DOX-JQ1@Gel. Meanwhile, no mice survived in any untreated and empty Gel group after 1 month. Although JQ1 can prolong the survival rate of tumor-bearing mice, it fails to inhibit the growth of tumors effectively. To further confirm the function of gel scaffold, free DOX and JQ1 were also intratumorally injected into 4T1-Luc tumor-bearing mice. As shown in Additional file 1: Fig. S7A–C, the combination of free drugs did not inhibit the growth of the tumor, and 20% of the mice were dead by day 6. After 2 weeks, almost all of the mice were dead with severe lung metastases. Therefore, the DOX-JQ1@Gel can combine advantages of JQ1 and DOX that can control not only tumor growth through combination chemoimmunotherapy but also prolong the survival of tumor-bearing mice. Furthermore, lung and tumors were harvested and analyzed via pathological H&E staining 7 days after treatments. As shown in Fig. 3D, lung metastases nearly occurred in control groups but not in the DOX-JQ1@Gel-treated group. Tumors in the DOX-JQ1@Gel-treated group also nearly disappeared compared with those in the control group.

**Fig. 4:**
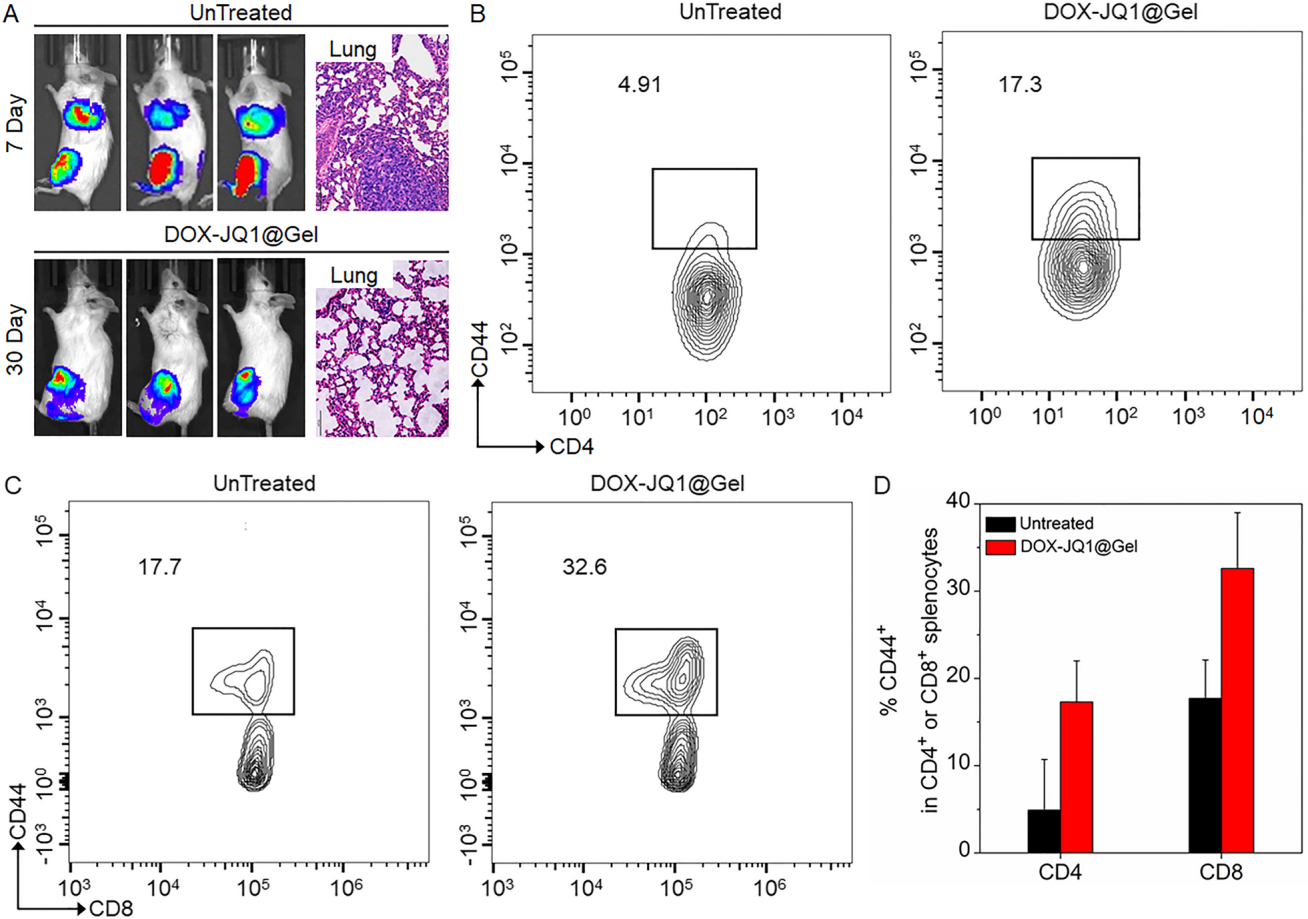
The inhibition mechanism of lung metastasis to local DOX-JQ1@Gel implantion. **A** In vivo bioluminescence imaging of 4T1-Luc breast cancer mice after treatment 7 and 30 days. Representative H&E staining of lungs collected from untreated and DOX-JQ1@Gel-treated mice after treatment 7 and 30 days. Splenocytes isolated from tumor-bearing control and treated mice were analyzed for the presence of CD4^+^CD44^+^ (**B**) and CD8^+^CD44^+^ (**C**) memory T cells. **D** Corresponding quantification of CD4 and CD8 memory T cells in splenocytes

Tumor cell signals were unclear in mouse lungs after 30 days of DOX-JQ1@Gel treatment, as confirmed by the pathological analysis (Fig. 4A). Treated mice and splenocytes were isolated from tumor-bearing control and analyzed to verify the presence of memory T cells and explore the inhibition mechanism of lung metastasis [33, 34]. Representative flow cytometry analysis demonstrated that CD8^+^CD44^+^ and CD4^+^CD44^+^ memory T cells significantly enhance in DOX-JQ1@Gel-implanted mice (Fig. 4B, C). Corresponding quantification results indicated that the respective CD8^+^CD44^+^ and CD4^+^CD44^+^ memory T cells of JQ1@Gel-implanted mice increase around 3.5 and 1.8 times compared with the untreated group. Moreover, toxic effects must also be considered in combination therapies [35]. Silk, chitosan, and PEG present high biocompatibility and are eliminated from the body. As shown in Additional file 1: Fig. S10, histology analysis of mouse organs after 45 days of treatment showed no evident abnormality or damage in organs.

### DOX-JQ1@Gel for prevention of distant metastases

**Fig. 5:**
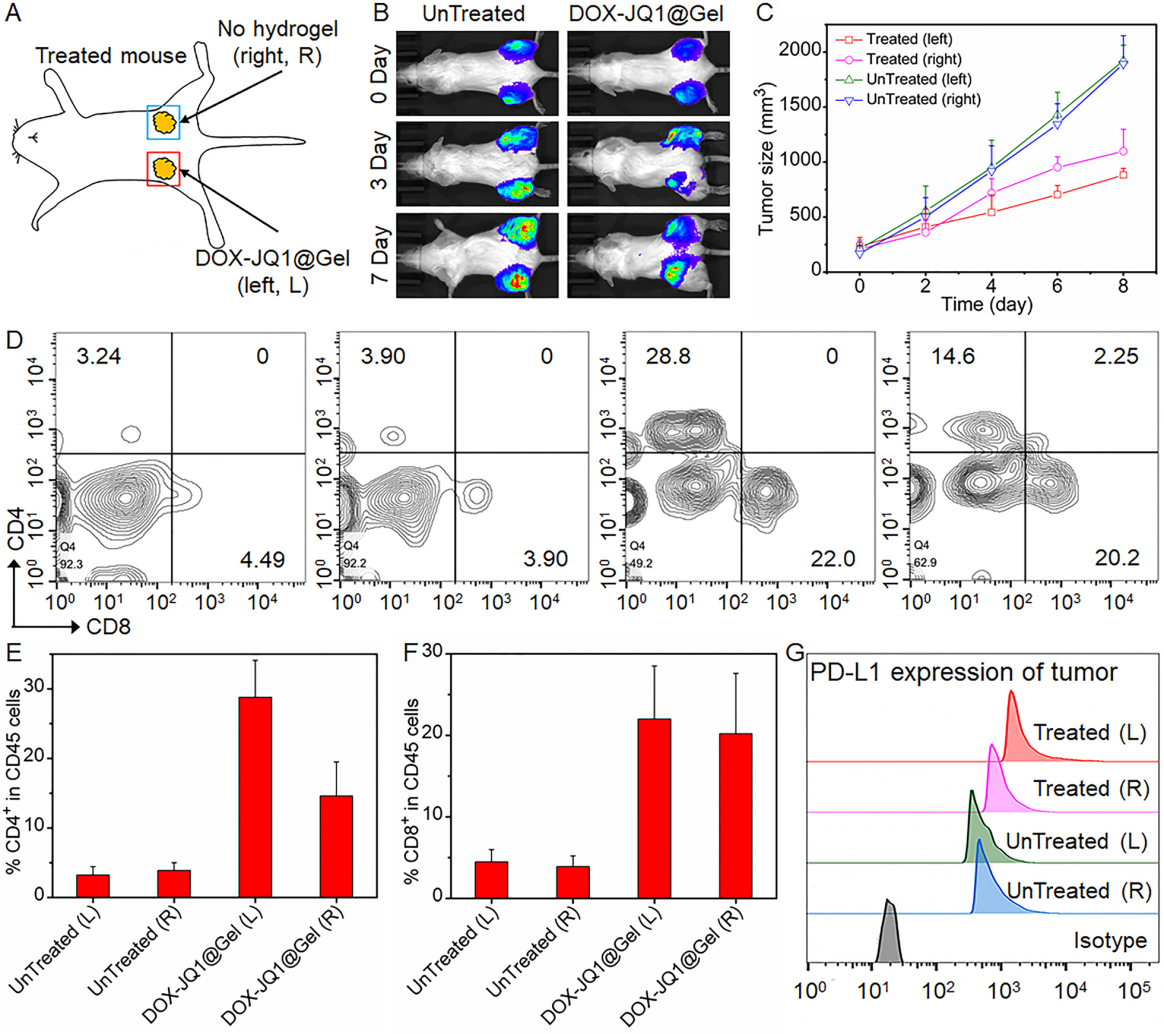
Local gel scaffold for abscopal anticancer immune response. **A** Mice were inoculated with tumor cells in the right and left flanks. Control mice were untreated, whereas treated mice were implanted with DOX-JQ1@Gel only on the left flank. **B** In vivo bioluminescence imaging of 4T1-Luc breast cancer in response to local DOX-JQ1@Gel treatment. **C** Left and right tumor growth curves on day 10 in untreated and treated mice. **D** Representative flow cytometry analysis of CD4^+^ and CD8^+^ T cells in tumors of control and treated mice. **E**, **F** Corresponding quantification results of CD4^+^ and CD8^+^ T cells in tumors of control and treated mice. **G** PD-L1 expression of tumor cells of the control and treated mice

Inoculation of tumor cells was conducted on the contrary site of the primary tumor where the DOX-JQ1@Gel was implanted to evaluate if local delivery of the DOX-JQ1@Gel causes systemic immune responses. As shown in Fig. 5A, the tumor on the left is injected with DOX-JQ1@Gel and the control is presented on the right. The DOX-JQ1@Gel-treated and contralateral tumor grew slowly within 1 week of dosing according to bioluminescence imaging and tumor growth curves shown in Fig. 5B, C. The tumor size significantly decreased in DOX-JQ1@Gel-treated and opposite tumor sites (Fig. 5D) and corresponding enhanced infiltration of CD4^+^CD8^+^ T cells compared with those of untreated mice (Fig. 5F). Furthermore, PD-L1 was upregulated by tumor cells at the two tumor sites in DOX-JQ1@Gel-treated tumors compared with untreated mice (Fig. 5G). The local systemic distribution of cytokines from the DOX-JQ1@Gel implant can explain this situation [36]. In addition, CD4^+^ and CD8^+^ T cells in the spleen shown in Additional file 1: Fig. S10 were also analyzed, and CD8^+^ T cells in the spleen of JQ1@Gel-treated mice were significantly higher than those in the untreated group.

## Discussion

The in situ-formed hydrogel scaffold involving a pH-sensitive scaffold can deliver DOX and JQ1 and show significant kinetic characteristics in tumor-bearing mice locally, thereby promoting the immune-mediated tumor rejection and immunogenic tumor phenotype in this study. Previous studies indicated that the remarkably improved expression of PD-L1 in tumor cells due to chemotherapy leads to PD-L1-mediated T cell exhaustion [37]. Thus, we assumed that the efficacy of anticancer can be enhanced by the ICB inhibitor with distinct kinetics and local administration of chemotherapeutics. The synthesis and loading of a pH-responsive silk/chitosan scaffold was conducted with DOX and JQ1 to realize cascade therapy at the tumor site given that low pH is prevalent in TME. DOX and JQ1 were released from the pH-responsive hydrogel in a pH-dependent manner when in situ construction was performed.

We observed downregulation of PD-L1 expression in TILs and tumor cells exposed to the DOX-JQ1@Gel. The decrease of tumor-infiltrating MDSCs due to the DOX-JQ1@Gel benefitted the dysfunction of the effect or T cells. Intratumoral MDSCs are generally depleted after the implantation of DOX-JQ1@Gel because DOX restrains intratumoral MDSCs. Similarly, the DOX-JQ1@Gel also induced the improvement of T cell infiltration and reduced CD206 expressed by TAMs. However, mice treated with empty hydrogel demonstrated a decrease in TAMs. As a result, the pH-responsive scaffold can be a reservoir for controlling the release of therapeutic drugs while acting as a scavenger of H^+^ in the TME to improve the curative effect. An immunogenic tumor phenotype was induced by the DOX-JQ1@Gel, and the activity of JQ1 promoted the regression of tumors in the 4T1 breast tumor mouse models with low PD-L1 levels. Local therapy created a systemic anticancer immune response that can inhibit tumor growth distantly. The combined chemoimmunotherapy strategy proposed in this study can jointly promote the treatment of poorly immunogenic tumors and reduce systemic toxicities. Although ICB is tolerable, the combined therapy could enhance the opportunity of side effects [38]. Toxicity was unclear in the group with scaffolds loaded by drugs in this study. Toxicity when using scaffolds in the long term should be assessed thoroughly as for more translation for clinical applications. Furthermore, doses and treatment frequencies of combination drugs can be investigated and optimized.

## Conclusion

In conclusion, a combined chemoimmunotherapy strategy was developed on the basis of the controlled release of ICB inhibitor and chemotherapeutic agent derived from a TME-responsive hydrogel scaffold. Immunogenic phenotypes in tumors were generated by DOX and JQ1 released by the hydrogel scaffold. The pH-responsive gels could be used as a reservoir to tune the release of therapies. As expected, this strategy can treat low-immunogenic tumors with poor responses to ICB. This strategy may also be used to overcome metastatic tumors and prevent the recurrence of tumors given that the local scaffold implantation is beneficial to T cell memory formation and systemic immune responses.

## Materials and methods

### Reagents and materials

Solvents and reagents bought from commercial suppliers were used without further purification unless otherwise specified. DOX and JQ1 were obtained from Amresco. The cell counting kit-8 assay (CCK-8), penicillin–streptomycin solution (100X), and trypsin were bought from Beyotime Institute of Biotechnology. PEG and FBS were obtained from Sinopharm Chemical Reagent Co., Ltd. and Biological Industries, respectively. All media were purchased from Corning Cellgro. Other chemicals were obtained from Aladdin (Shanghai, China). Human umbilical vein endothelial cells (HUVEC) and mouse macrophage cell line (RAW264.7) and were supplied by Cell Bank of Shanghai, Chinese Academy of Sciences (Shanghai, China). Four-week-old BALB/c mice (20 g) were bought from Shanghai SLAC Laboratory Animal Co., Ltd. and then fed under a 12 h light/dark cycle.

### Characterization

DF-PEG was characterized via ^1^H NMR. Morphology of blank and drug-loaded gels was characterized using a Cryo-SEM (JEOL 7600F). VERTEX 70 spectrometer (Bruker) was utilized to record Fourier transform infrared (FT-IR) spectra.

### Synthesis of DF-PEG

The preparation of DF-PEG is referred to in a previous study. *p*-Formyl benzoic acid (0.98 g, 6.52 mmol), DMAP (0.050 g), and *N*,*N*′-dicyclohexylcarbodiimide (1.68 g, 8.15 mmol) were added in a solution of PEG2000 (3.26 g, 1.63 mmol) in dry THF (100 mL). The mixture was stirred at a temperature of 25 °C for 24 h in nitrogen atmosphere until a white solid was obtained and filtered. The mixture was then precipitated using diethyl ether. The crude product was dissolved repeatedly in THF and then precipitated in diethyl ether three times to obtain the white solid. The product was dried at room temperature under decreased pressure, which obtained 3.12 g of DF-PEG in a yield of 82.9%.

### Preparation of silk-chitosan hydrogel

Hydrogels in this study were prepared as follows. A chitosan/silk solution (3%, w/w) was formed by dissolving chitosan into silk phosphate buffer saline (PBS). DF-PEG (1.0 g) was dissolved in 4.0 g of PBS to form the DF-PEG solution (20%, w/w). The same volume of silk and DF-PEG solution in PBS was injected into a vial by adopting a commercially available 3 mL 1:1 FibriJet® Applicator Assembly (Shanghai Misawa Medical Industry Co., Ltd.). The precursor mixture solution in the vial was bathed in water at 37 °C to record the gelation time through vial inverting.

### pH-responsive ability of the injectable hydrogel

Experiments were conducted to demonstrate the pH-responsive ability of the hydrogel upon stimulus as follows. The gel was prepared and a small amount of DOX was added to improve tracking. Liquefaction was performed by adding saturated HCl solution (60 μL, 12 M) to the gel within 15 min via vortexing. The hydrogel was regenerated in ~ 60 s by adding concentrated NaOH solution (60 μL, 12 M). Sol–gel transitions were regulated using HCl or NaOH solution and repeated at least five times.

### DOX and JQ1 release from silk hydrogels

Prepared samples were maintained at a temperature of 37 °C for 6 h. Buffer solutions at pH = 7.4 (10 mL), pH = 6.0 (10 mL), and pH = 5.0 (10 mL) were then added to three vials. The released DOX was determined with UV–Vis photospectrometry and the JQ1 release was analyzed via HPLC.

### Biocompatibility evaluation

NIH-3T3 or 4T1 cells were placed in a 96-well plate (5 × 10^3^ cells/well) in 100 µL of DMEM medium containing 1% l-glutamine, 10% FBS, streptomycin (100 μg/mL), antibiotics (100 IU/mL), and penicillin (100 IU/mL) for 24 h. The medium was removed. Freshly prepared I-BSA NPs with a series of concentrations (100 μL) were placed in the plate. Cells were then cultured for 24 h, and the medium was aspirated, washed with PBS, and replaced by 10 μL of CCK-8 solution and 90 μL of fresh medium. Cells were cultured for 4 h to detect the absorbance at 405 nm by adopting a microplate reader (Synergy NEO, BioTek). Each treatment group was provided with six wells. Data are presented as average ± SD.

### In vivo tumor models

Mice were investigated for 14 days to detect anticancer effects in mouse models when 1 × 10^6^ of luciferase-tagged 4T1 tumor cells were transplanted into the right side of mice. Subjects were randomly divided into several groups (*n* = 5) after weighing. Different formulations, including DOX@Gel, JQ1@Gel, DOX-JQ1@Gel, and hydrogels, were peritumorally implanted into mice. The bioluminescence signal of cancer cells was used to monitor the tumor burden. Images were recorded with an IVIS Lumina imaging system (Caliper). A digital caliper was applied to measure tumors, and the tumor volume (mm^3^) was calculated as (long diameter × short diameter^2^)/2.

### Cytokine detection

LEGENDplex Mouse Th1 Panel multiple assay (catalog no. 740025, BioLegend) was adopted according to the manufacturer’s instructions to measure plasma concentrations of IL-6 and IFN-γ. The plasma of mice was then collected before hydrogel implantation and 2 days after the implantation.

## Supplementary Information

**Additional file 1: Figure S1.** Morphology changes of hydrogels in pH 5.0 and pH 7.4 buffer solution over 48 h. **Figure S2.** Representative Cryo-SEM image of gel scaffold treated by pH 5.0 buffer solutions. **Figure S3.** Cytotoxicity of gel raw materials, empty gels and gel extracts to normal cells (3T3) and breast cancer 4T1 cells. **Figure S4.** In vivo biodegradability of DOX-JQ1@Gel was verified by real-time fluorescence imaging (A) and quantitative analysis (B). The main organs for fluorescence imaging (C) and quantitative analysis (D) were obtained after 120 h in vivo imaging of DOX-JQ1@Gel treated mice. **Figure S5.** Representative flow cytometric analysis of T cell infiltration within the tumor (CD45^+^, CD4^+^, CD8^+^), MDSCs (CD11b^+^Gr-1^+^), M2 macrophages (CD206^+^) in F4/80^+^ CD11b^+^ CD45^+^ cells, and regulatory T cells (Tregs, FOXP3^+^ in CD4^+^ CD25^+^ CD45^+^ cells). **Figure S6.** Related immunogenic death indicators were evaluated in vitro, including ATP and CRT. With the increase of DOX concentration, gradually increased expression of ATP and CRT indicates that immunogenic death is concentration-dependent. **Figure S7.** Local DOX and JQ1 for inhibition of 4T1-Luc breast cancer growth in vivo (n = 5). (A) In vivo photograph of the mice baring 4T1-Luc breast cancer treated with free DOX and JQ1. (B) Time dependent tumor growth kinetics and growth curves were stopped when the animal died. (C) A photograph of a representative lung at day 7 after treatments. **Figure S8.** Circulating cytokines (IFN-γ, IL-6) expression from the 4T1 tumor xenograft treated with Gel, DOX@Gel, JQ1@Gel or DOX-JQ1@Gel. **Figure S9.** (**A**) Representative flow cytometry analysis of T cells in splenocytes of untreated and DOX-JQ1@Gel-treated mice, and corresponding quantification results (**B**). (**C**) Representative flow cytometry analysis of CD4^+^ and CD8^+^ T cells in splenocytes of untreated and DOX-JQ1@Gel-treated mice, and corresponding quantification results (**D**). **Figure S10.** Pathological H&E staining of heart, liver, spleen and kidney from the treated and control mice at day 21 after treatments.

## Data Availability

All of the material is owned by the authors and no permissions are required.
