# Supplementary material for: Injectable pH-responsive hydrogel for combinatorial chemoimmunotherapy tailored to the tumor microenvironment
Source: J Nanobiotechnology. 2022 Aug 11;20:372. doi: 10.1186/s12951-022-01561-z (PMC9367026; doi:10.1186/s12951-022-01561-z)
Supplement: Supplementary file 1 — Additional file 1: Figure S1. Morphology changes of hydrogels in pH 5.0 and pH 7.4 buffer solution over 48 h. Figure S2. Representative Cryo-SEM image of gel scaffold treated by pH 5.0 buffer solutions. Figure S3. Cytotoxicity of gel raw materials, empty gels and gel extracts to normal cells (3T3) and breast cancer 4T1 cells. Figure S4. In vivo biodegradability of DOX-JQ1@Gel was verified by real-time fluorescence imaging (A) and quantitative analysis (B). The main organs for fluorescence imaging (C) and quantitative analysis (D) were obtained after 120 h in vivo imaging of DOX-JQ1@Gel treated mice. Figure S5. Representative flow cytometric analysis of T cell infiltration within the tumor (CD45+, CD4+, CD8+), MDSCs (CD11b+Gr-1+), M2 macrophages (CD206+) in F4/80+ CD11b+ CD45+ cells, and regulatory T cells (Tregs, FOXP3+ in CD4+ CD25+ CD45+ cells). Figure S6. Related immunogenic death indicators were evaluated in vitro, including ATP and CRT. With the increase of DOX concentration, gradually increased expression of ATP and CRT indicates that immunogenic death is concentration-dependent. Figure S7. Local DOX and JQ1 for inhibition of 4T1-Luc breast cancer growth in vivo (n = 5). (A) In vivo photograph of the mice baring 4T1-Luc breast cancer treated with free DOX and JQ1. (B) Time dependent tumor growth kinetics and growth curves were stopped when the animal died. (C) A photograph of a representative lung at day 7 after treatments. Figure S8. Circulating cytokines (IFN-γ, IL-6) expression from the 4T1 tumor xenograft treated with Gel, DOX@Gel, JQ1@Gel or DOX-JQ1@Gel. Figure S9. (A) Representative flow cytometry analysis of T cells in splenocytes of untreated and DOX-JQ1@Gel-treated mice, and corresponding quantification results (B). (C) Representative flow cytometry analysis of CD4+ and CD8+ T cells in splenocytes of untreated and DOX-JQ1@Gel-treated mice, and corresponding quantification results (D). Figure S10. Pathological H&E staining of heart, liver, spleen and k [file 12951_2022_1561_MOESM1_ESM.doc]

Additional file 1

Injectable pH-Responsive Hydrogel for Combinatorial Chemoimmunotherapy Tailored to the Tumor Microenvironment

Jun Gu,^‡,‖^ Gang Zhao,^†,‖^ Jiangkun Yu,^†^ Pei Xu,^†^ Jiabin Yan,^‡^ Zhengshuai Jin,^‡^ Sheng Chen,^‡,*^ Yong Wang^†^, Leshuai W. Zhang,^†^ Yangyun Wang^†^,*

^†^State Key Laboratory of Radiation Medicine and Protection, School for Radiological and Interdisciplinary Sciences (RAD-X), Collaborative Innovation Center of Radiation Medicine of Jiangsu Higher Education Institutions, Soochow University, Suzhou 215123, China

^‡^The Affiliated Jiangsu Shengze Hospital of Nanjing Medical University, Suzhou 215228, China


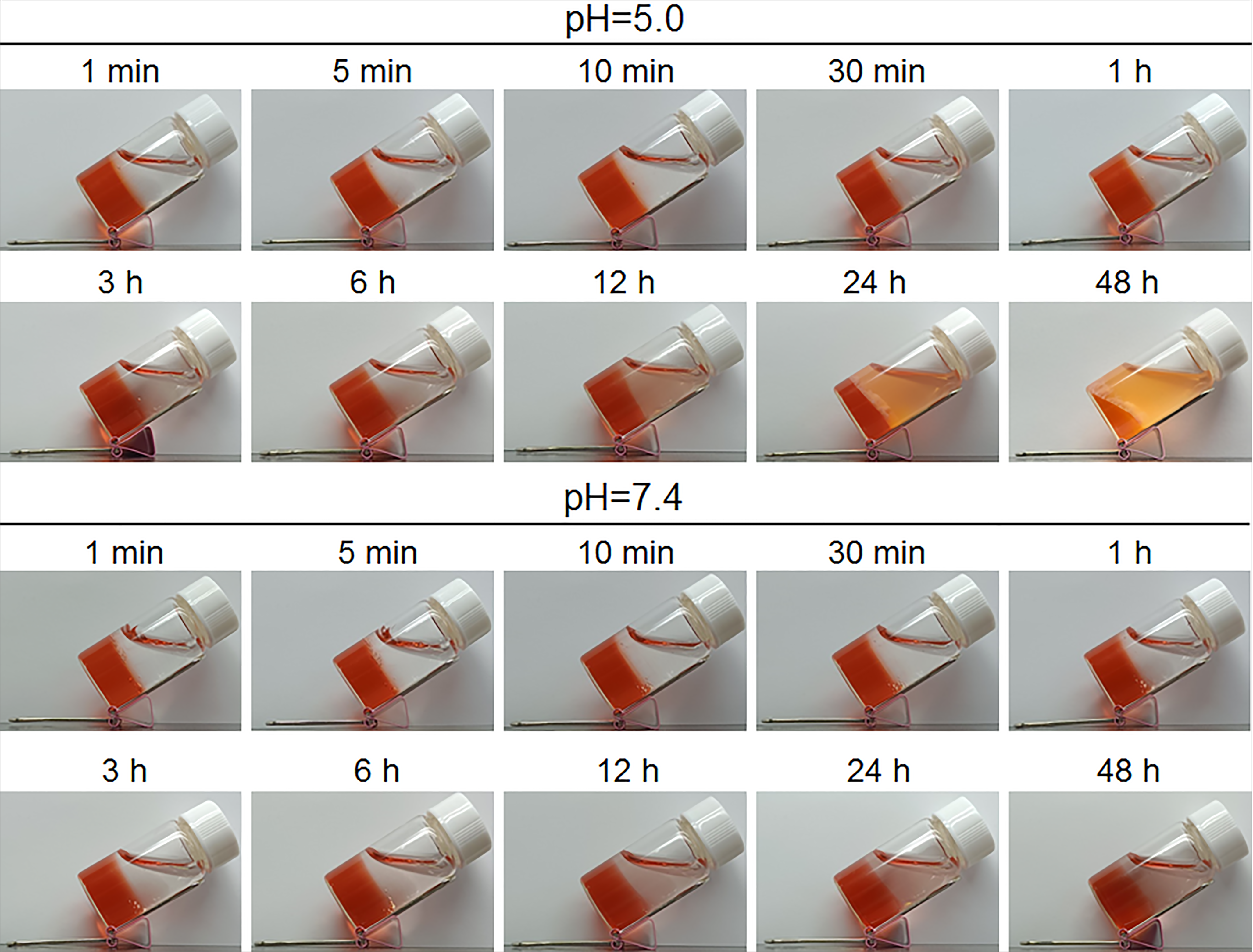


**Figure S1.** Morphology changes of hydrogels in pH 5.0 and pH 7.4 buffer solution over 48 hours.


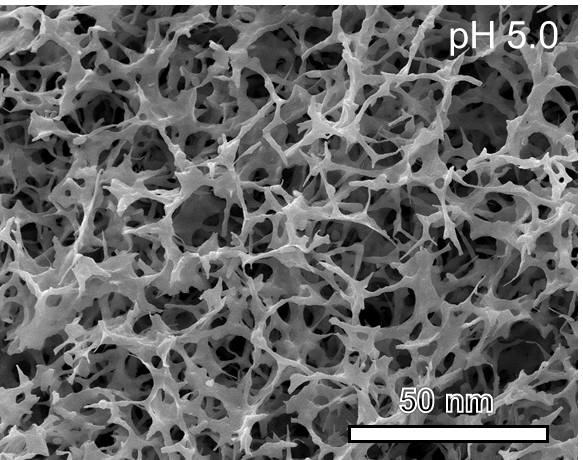


**Figure S2.** Representative Cryo-SEM image of gel scaffold treated by pH 5.0 buffer solutions.


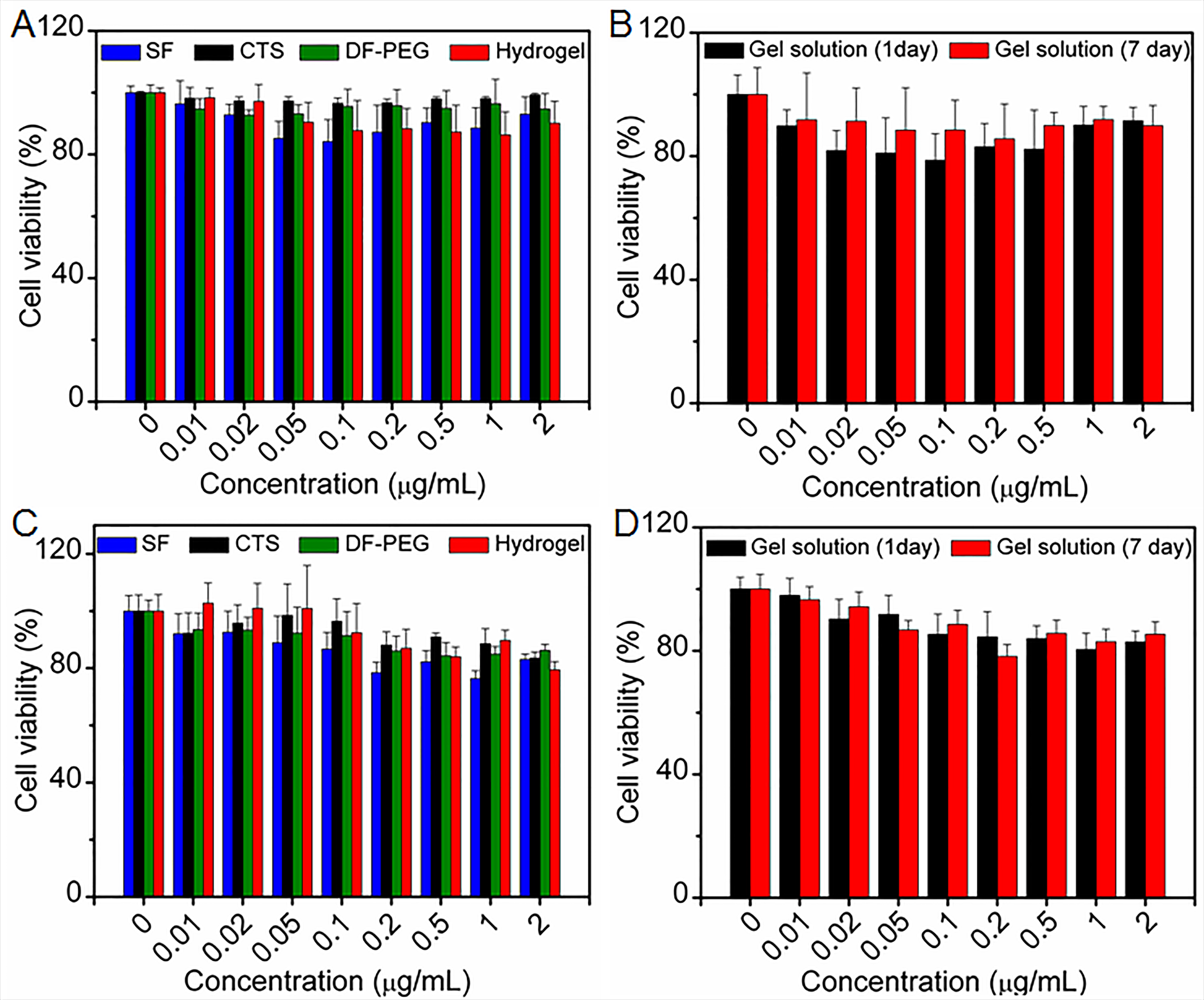


**Figure S3.** Cytotoxicity of gel raw materials, empty gels and gel extracts to normal cells (3T3) and breast cancer 4T1 cells.


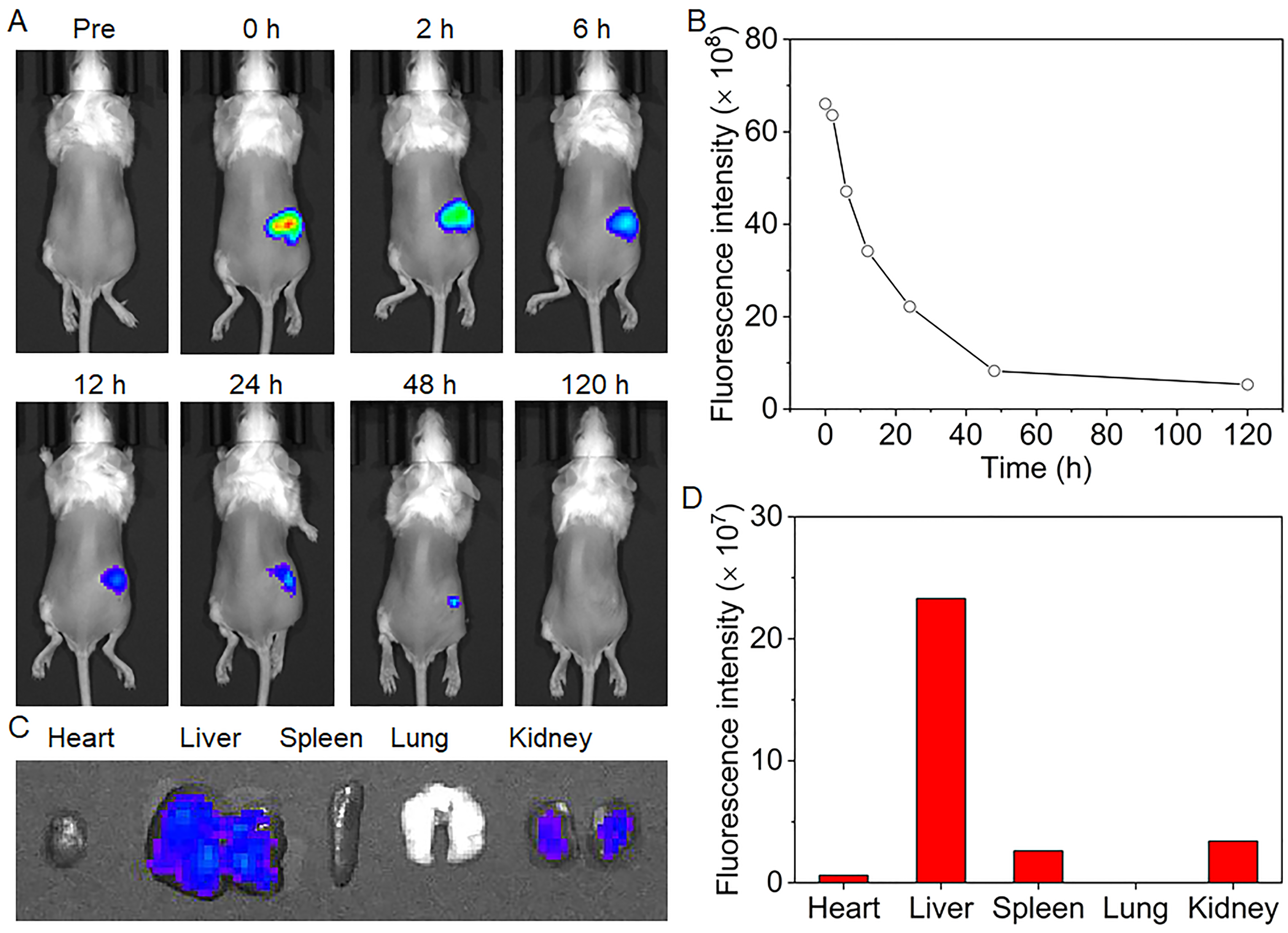


**Figure S4.** *In vivo* biodegradability of DOX-JQ1@Gel was verified by real-time fluorescence imaging (A) and quantitative analysis (B). The main organs for fluorescence imaging (C) and quantitative analysis (D) were obtained after 120 h in vivo imaging of DOX-JQ1@Gel treated mice.


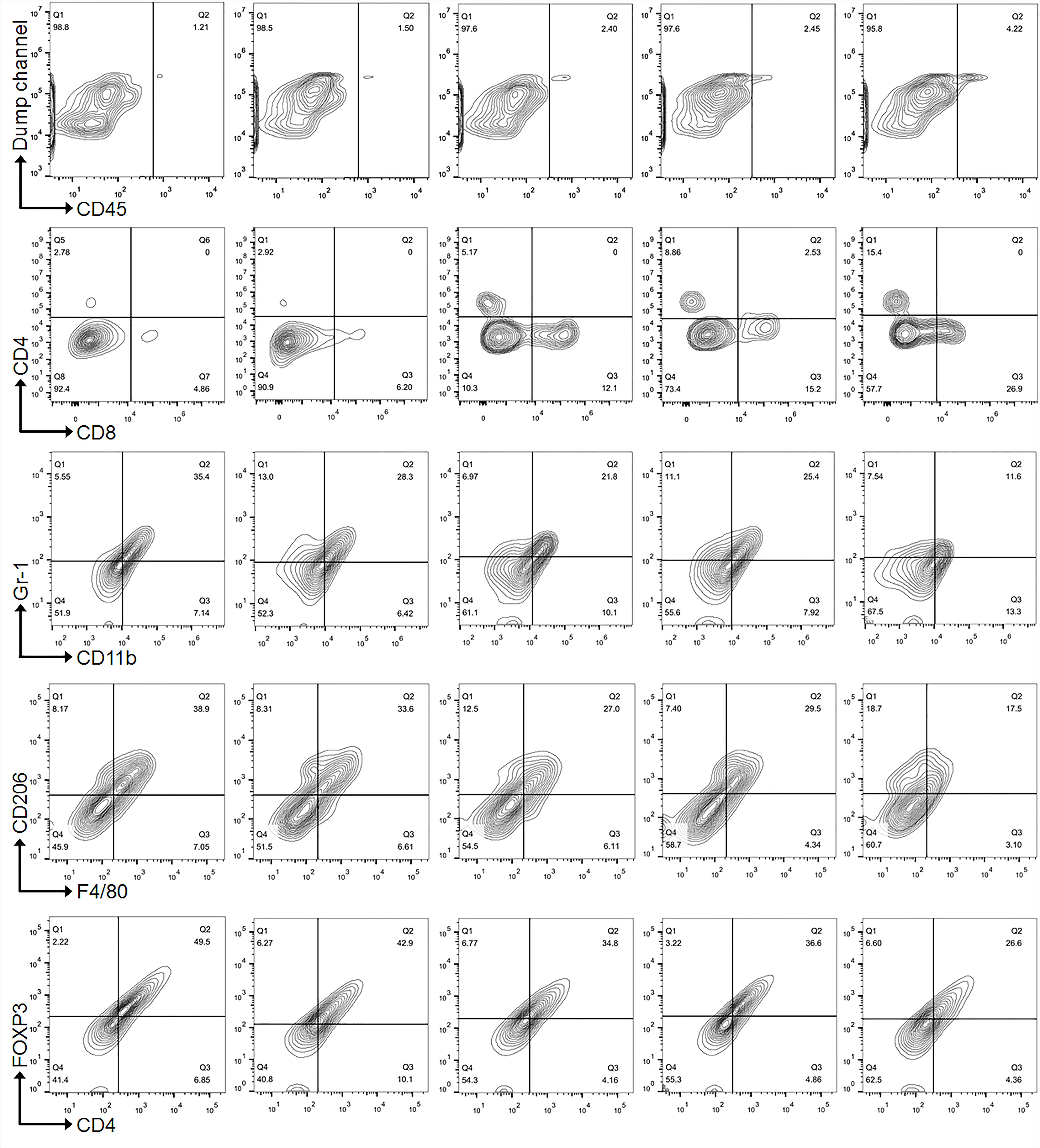


**Figure S5.** Representative flow cytometric analysis of T cell infiltration within the tumor (CD45^+^, CD4^+^, CD8^+^), MDSCs (CD11b^+^Gr-1^+^), M2 macrophages (CD206^+^) in F4/80^+^ CD11b^+^ CD45^+^ cells, and regulatory T cells (Tregs, FOXP3^+^ in CD4^+^ CD25^+^ CD45^+^ cells).


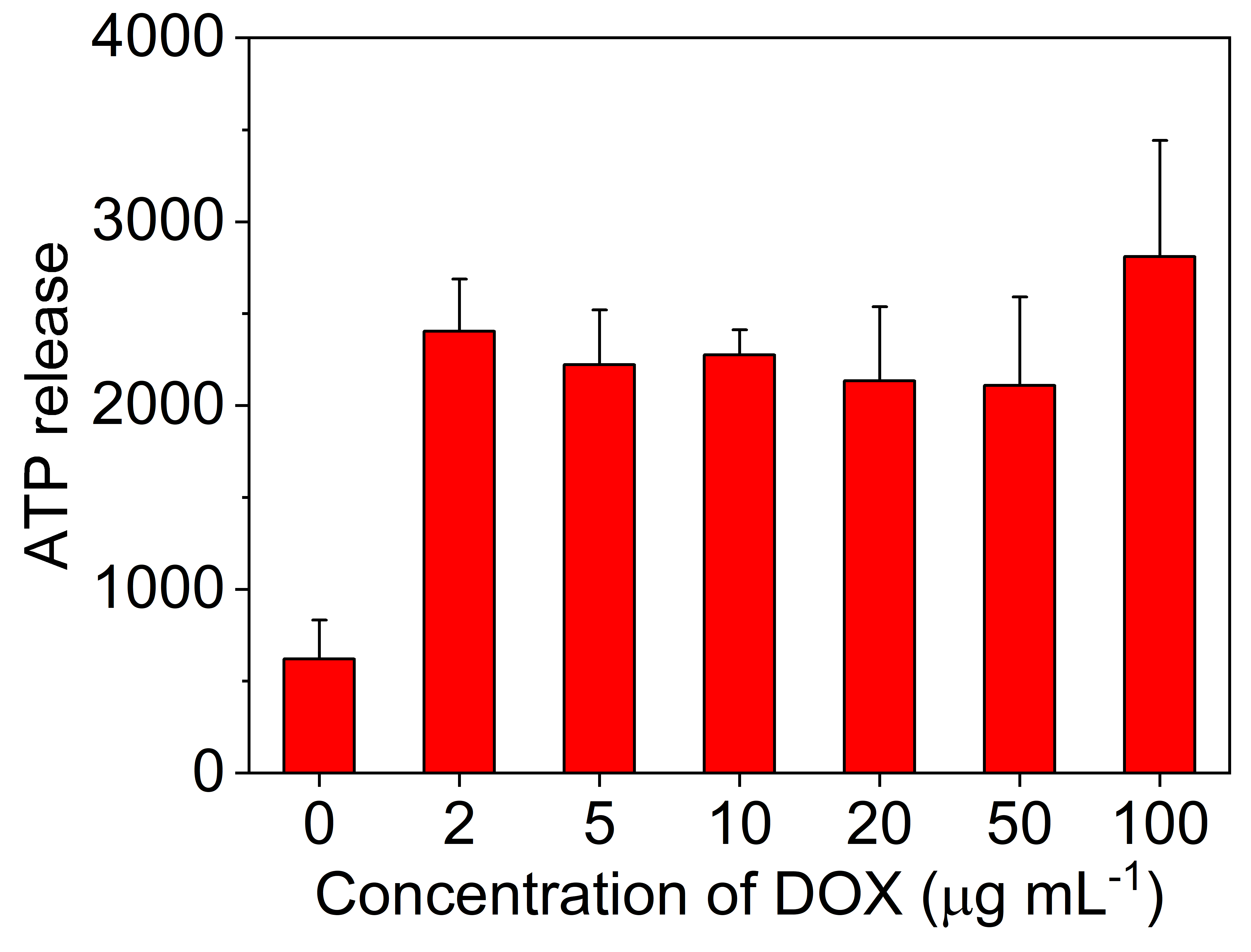

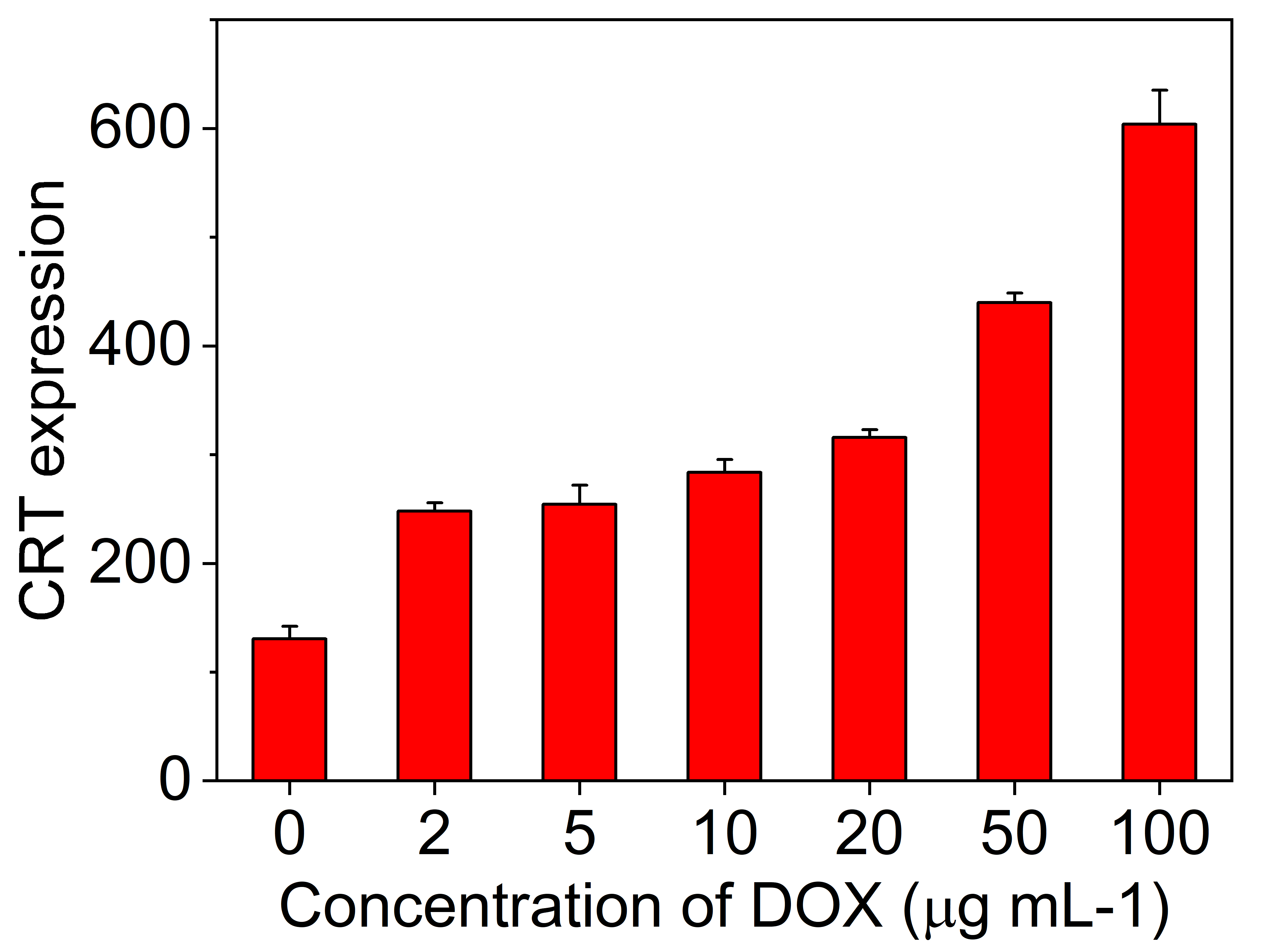


**Figure S6.** Related immunogenic death indicators were evaluated in vitro, including ATP and CRT. With the increase of DOX concentration, gradually increased expression of ATP and CRT indicates that immunogenic death is concentration-dependent.


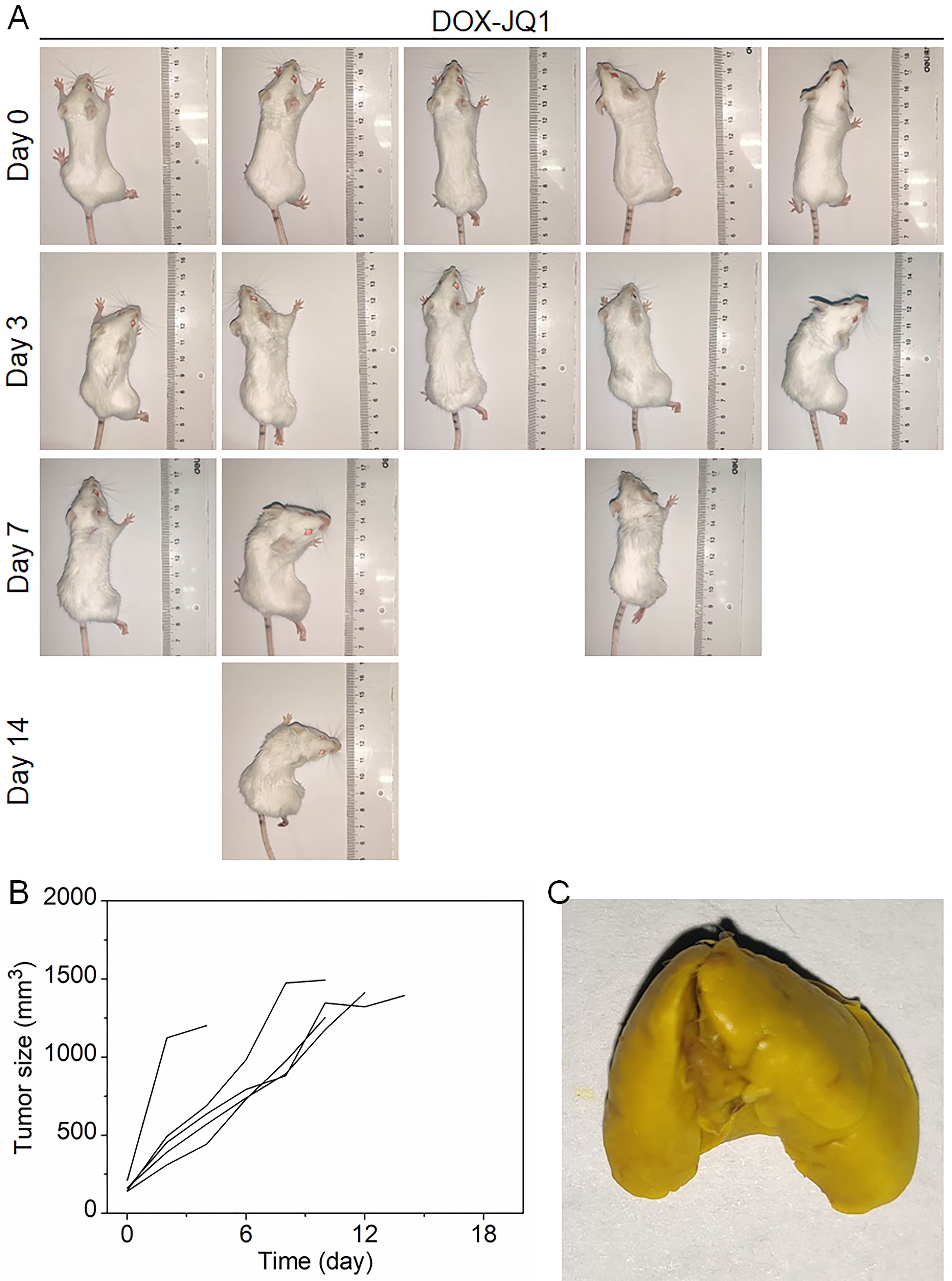


**Figure S7. Local DOX and JQ1 for inhibition of 4T1-Luc breast cancer growth in vivo (n=5).** (A) In vivo photograph of the mice baring 4T1-Luc breast cancer treated with free DOX and JQ1. (B) Time dependent tumor growth kinetics and growth curves were stopped when the animal died. (C) A photograph of a representative lung at day 7 after treatments.


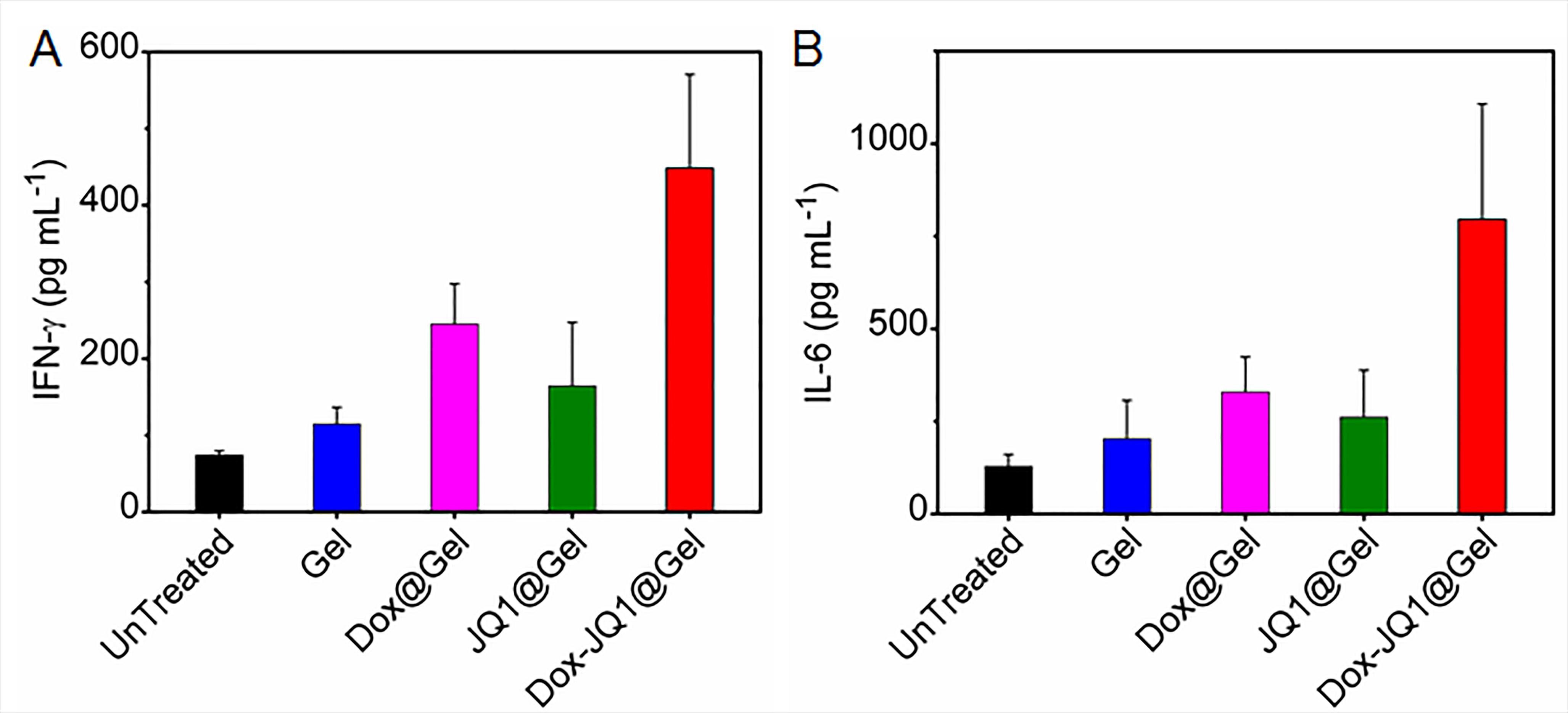


**Figure S8.** Circulating cytokines (IFN-γ, IL-6) expression from the 4T1 tumor xenograft treated with Gel, DOX@Gel, JQ1@Gel or DOX-JQ1@Gel.


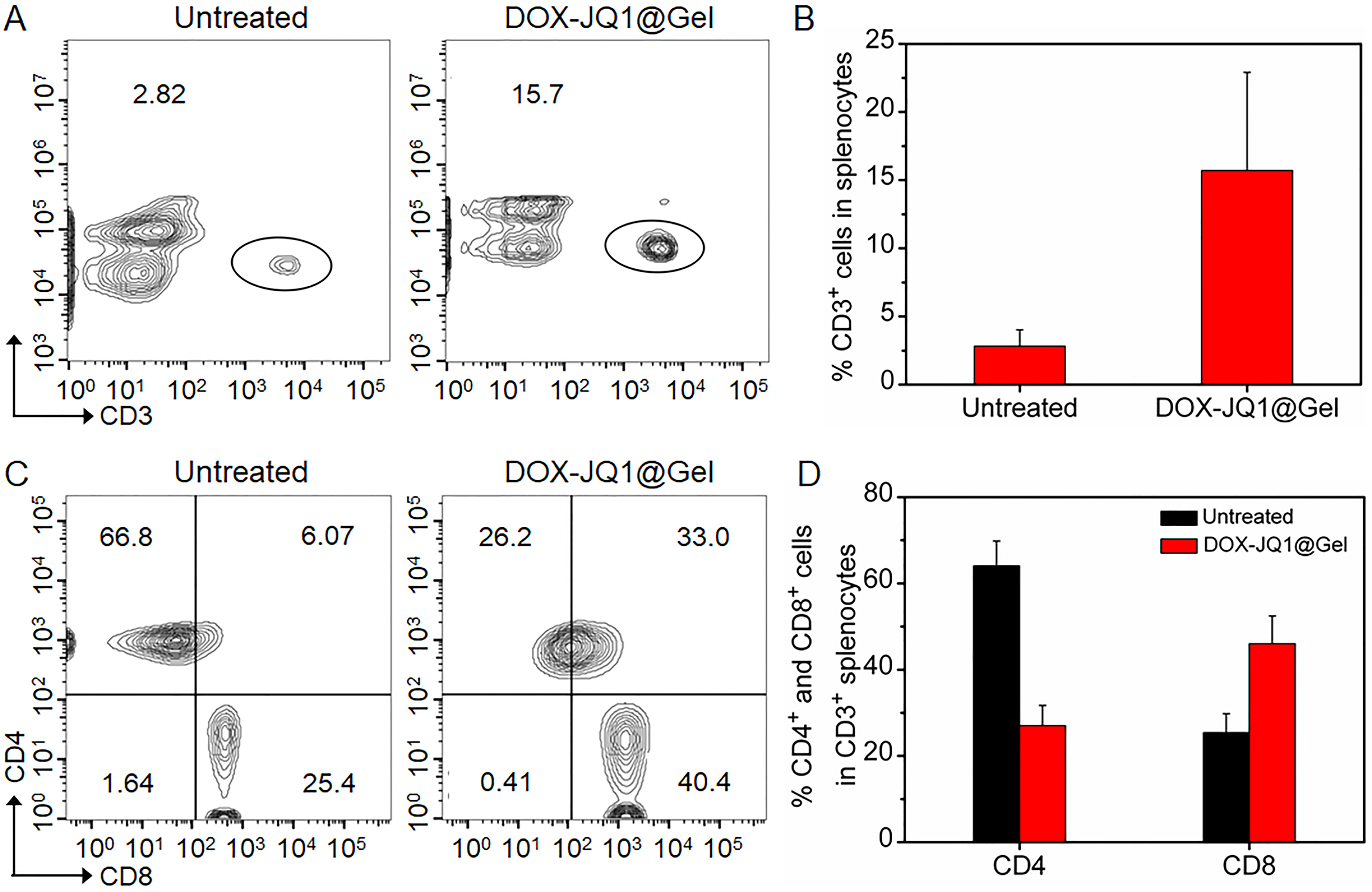


**Figure S9.** (**A**) Representative flow cytometry analysis of T cells in splenocytes of untreated and DOX-JQ1@Gel-treated mice, and corresponding quantification results (**B**). (**C**) Representative flow cytometry analysis of CD4^+^ and CD8^+^ T cells in splenocytes of untreated and DOX-JQ1@Gel-treated mice, and corresponding quantification results (**D**).

**Figure S10.** Pathological H&E staining of heart, liver, spleen and kidney from the treated and control mice at day 21 after treatments.
